# Nationwide study on the prevalence of rheumatoid factor and anticitrullinated peptide positivity and their contribution to rheumatoid arthritis diagnosis

**DOI:** 10.55730/1300-0144.5872

**Published:** 2024-10-07

**Authors:** Hasan SATIŞ, Abdulsamet ERDEN, Emre BİLGİN, Gizem AYAN, Berkan ARMAĞAN, Duygu TECER, Alper SARI, Umut KALYONCU, Murat ÇAĞLAYAN, Mustafa Mahir ÜLGÜ, Mustafa Okan AYVALI, Naim ATA, Şuayip BİRİNCİ

**Affiliations:** 1 Division of Rheumatology, Department of Internal Medicine, Abdurrahman Yurtaslan Oncology Research and Training Hospital, University of Health Sciences, Ankara, Turkiye; 2 Division of Rheumatology, Department of Internal Medicine, Faculty of Medicine, Gazi University, Ankara, Turkiye; 3 Division of Rheumatology, Department of Internal Medicine, Faculty of Medicine, Hacettepe University, Ankara, Turkiye; 4 Division of Rheumatology, Department of Internal Medicine, Ankara Bilkent State Research and Training Hospital, University of Health Sciences, Ankara, Turkiye; 5 Division of Rheumatology, Department of Internal Medicine, Gülhane Research and Training Hospital, University of Health Sciences, Ankara, Turkiye; 6 Division of Rheumatology, Department of Internal Medicine, Ankara Etlik State Research and Training Hospital, University of Health Sciences, Ankara, Turkiye; 7 Department of Biochemistry, Ankara Bilkent State Research and Training Hospital, Ankara Yıldırım Beyazit University, Ankara, Turkiye; 8 Department of Health Information Systems, Republic of Turkey, Ministry of Health, Ankara, Turkiye; 9 Department of Strategy Development, Republic of Turkey, Ministry of Health, Ankara, Turkiye; 10 Republic of Turkey, Ministry of Health, Ankara, Turkiye

**Keywords:** Autoantibody, rheumatoid factor, anticitrullinated peptide, nationwide, rheumatoid arthritis

## Abstract

**Background/aim:**

Rheumatoid factor (RF) and anticitrullinated peptide (anti-CCP) are specific for rheumatoid arthritis (RA) diagnosis. However, they could be positive in other diseases and even in healthy populations. The aim was to investigate the prevalence of positive RF and anti-CCP antibodies in persons admitted to hospital for any reason and on a national scale.

**Materials and methods:**

The National Electronic Health Database, which contains the clinical records of over 80 million people, was used to design this multicenter, retrospective cohort study. The subjects included in the study were divided into age groups according to 10-year periods. RA cases were identified using ICD-10 codes that included M05, M06, M08, and their subgroups. RF and anti-CCP positivity were evaluated in terms of their contribution to the risk of being diagnosed with RA, with the change according to age and sex.

**Results:**

During the 1.1.2018–31.12.2021 period, 13,918,072 RF tests were performed in 11,849,440 people, whereas 1,183,607 anti-CCP tests were performed in 1,020,967 people. Moreover, 797,089 people had both tests performed at least once. The RF positivity rate in patients who only requested RF tests was 14.72% and it was 35.04% for anti-CCP positivity in those who only requested anti-CCP tests. The rate of concomitant RF and anti-CCP positivity was 22.56%. An RA diagnosis was made in 27.8% of RF-positive people, 39.73% of anti-CCP-positive people, and 56.6% of co-RF and anti-CCP-positive people. RF positivity and concomitant RF and anti-CCP positivity increased with age and were more common in females.

**Conclusion:**

RF and anti-CCP positivity may be seen in a healthy population with female predominance. As age increases, the risk of RF positivity rises, but anti-CCP positivity does not change. Concomitant RF and anti-CCP positivity shows the highest risk of RA development with respect to either antibody positivity alone.

## 1. Introduction

Rheumatoid arthritis (RA) is a systemic disease that causes symmetrical polyarthritis particularly involving hand and foot joints [1]. The disease prevalence ranges between 0.5% and 1.0% in Europe, North America, and Japan, with a female predominance [2]. However, relatively few epidemiological studies have been carried out in Türkiye or internationally to determine the prevalence of RA and rheumatoid factor (RF) and anticitrullinated peptide (anti-CCP) positivity [3–5].

RA is diagnosed by comprehensively evaluating clinical symptoms, imaging findings, and laboratory markers, such as RF and anti-CCP [6]. RF and/or anti-CCP positivity could occur years before the development of the disease and are associated with a worse prognosis [7]. These autoantibodies are highly specific for RA, although their presence does not rule out other autoimmune diseases like SLE [8]. RF and anti-CCP are specific for RA diagnosis and depending on the method of detection and the cut-off value used, 50%–90% of RA patients have RF positivity, whereas 55%–91% of RA patients have anti-CCP positivity [1,4,7]. Although both antibodies have excellent sensitivity for RA diagnosis, some studies suggested that the diagnostic accuracy of both anti-CCP antibody and IgM–RF positivity was not markedly better than that of anti-CCP antibody positivity alone [9]. On the other hand, both antibodies can be seen in nonrheumatologic conditions as well as in the healthy population [4,10,11].

Several studies have focused on the positivity of RF within the general population. The prevalence varies between 2.8% and 21.6% depending on the population type, with female dominance [3,5,10,12–14]. RF positivity also increases with age and is seen in 20% of people aged 65 and over [15]. On the other hand, anti-CCP positivity in the general population varies between 0.4% and 2.8% depending on ethnicity, with a female predominance [3,4,13,16,17]. All these studies, the largest of which involved 40,000 people, were conducted in healthy populations or blood donors. No studies have been done in individuals with any complaints.

The objective of the present study was to determine cost, request numbers from different levels of care, and positivity rates of RF and anti-CCP antibodies in persons admitted to hospital for any reason on a national scale. In addition to the diagnosis of RA and the condition of antibodies, the change in positivity of autoantibodies with sex and age was also assessed.

## 2. Materials and methods

### 2.1. Study design and data source

The Turkish Ministry of Health National Electronic Database (e-Pulse) was used to design this multicenter, retrospective cohort study. Since 2014, the Ministry of Health has been establishing health data warehouses covering the entire country. In 2015, the Ministry of Health established the e-Pulse system as a national health information system, to which only authorized individuals and institutions have access and which has wide bandwidth and covers all of the country [18]. As Türkiye has a universal system called General Health Insurance, all Turkish residents can receive medical services free of charge through the Social Security Institution. All data for the study were obtained from the abovementioned central national database, which is under the control of the Turkish Ministry of Health. The Ministry of Health presents services using big data technology, and these systems are integrated together: e-Pulse and the National Healthcare Information System (NHIS).

The e-Pulse system contains the clinical records of over 80 million people in Türkiye, which include demographic characteristics, laboratory results, drug history, and comorbidities. To examine the change in autoantibody positivity with age, the people included in the study were divided into age groups according to 10-year periods, with the youngest age group being 20 years and younger and the largest age group 71 years old and above.

Our study was conducted according to the Declaration of Helsinki and was approved by the Ministry of Health Ethical Board (95741342-020/27112019).

### 2.2. RA diagnosis and autoantibody positivity

The study period ranged from 1 January 2018 to 31 December 2021. RA cases were identified using ICD-10 codes that included M05, M06, M08, and their subgroups. Patients who were entered with those ICD 10 codes twice at least 3 months apart were considered to have RA. ELISA laboratory results of RF and anti-CCP antibodies are given in IU/mL. If there was more than one RF or anti-CCP result for a patient, the highest titer was accepted as the value for that patient. The result for the antibody is considered positive if the result is higher than the laboratory upper limit of the normal test range of 30 IU for both RF and anti-CCP. An RF test costs 6.7 Turkish lira (TL) and an anti-CCP test costs 29.3 TL and the cost of RF testing was estimated as “cost per diagnosis of RA” and “cost per diagnosis of seropositive RA”.

### 2.3. Statistical analysis

SPSS for Windows (SPSS Inc., Chicago, IL, USA) was used for the statistical analyses. Variables were examined using visual and analytical methods to determine whether they were normally distributed. Categorical variables were shown as numbers and frequencies, with differences being analyzed by the chi-squared test. Continuous data that followed a normal distribution were described with mean and standard deviation and between-group comparisons were performed by independent samples t-test.

e-Pulse data were instantly transferred to Cloudera (Cloudera CDH, v. 6.3.2), a Hadoop-based big data environment for reporting and analysis. In the big data environment on Hadoop, Kudu table type storage is used for writing and updating data from live sources, Parquet type storage format for reporting data and tables to be queried analytically, and Apache Impala (v. 3.2.0) query for data recording and querying engine. Impala servers, where the data are kept, consist of a total of 97 clusters, as of September 2021, a total of 95 data nodes (holding data), and 2 coordinator nodes (distributing and collecting queries and requests to other servers). The CentOS 7 operating system is installed on all servers.

## 3. Results

### 3.1. RF and anti-CCP requests and positivity in the general population

#### 3.1.1. Rheumatoid factor requests

During the study period, 13,918,072 RF tests were performed in 11,849,440 people. Moreover, 3,479,518 RF tests per year were requested for 2,962,360 patients and for 403,402 (15.8%) people more than one RF test per year was requested. The mean age was 47.7 ± 7.9 and 67.4% of them were female. The estimated cost of the 13,918,072 RF tests was 94,502,615 TL and was 149.5 TL per RA diagnosis while 500 TL was spent per case of seropositive RA (Table).

#### 3.1.2. Anti-CCP requests

During the study period, 1,183,607 anti-CCP tests were performed in 1,020,967 people. In addition, 295,901 anti-CCP tests per year were requested for 280,037 patients and for 14,518 (5.2%) people more than one anti-CCP test per year was requested. The mean age was 47.9 ± 7.5 and 75.4% of them were female. Furthermore, 797,089 people had both tests performed at least once. The estimated cost of the 1,183,607 anti-CCP tests was 34,699,472 TL and was 55 TL per RA diagnosis while 183 TL was spent per case of seropositive RA.

#### 3.1.3. RF and/or anti-CCP positivity

**Figure 1:**
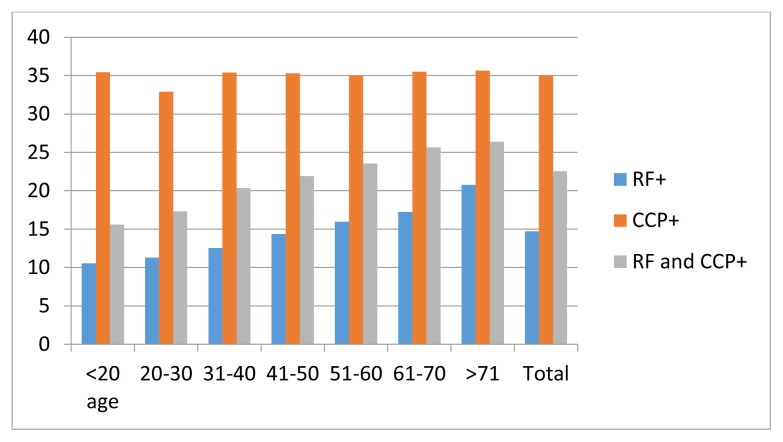
RF, anti-CCP, and concomitant RF and CCP positivity percentage with respect to age after requesting autoantibody tests in the general population.

The RF positivity rate in patients who only requested an RF test was 14.7% and it was 35.0% for anti-CCP positivity in those who only requested an anti-CCP tests. The rate of concomitant RF and anti-CCP positivity was 22.6% in patients who requested both. RF positivity and concomitant RF and anti-CCP positivity increased with age. In the youngest group, the RF positivity rate was 10.6% and the RF plus anti-CCP positivity rate was 15.6; it was 20.7% and 26.4% in the oldest group, respectively. Anti-CCP positivity was similar between age groups and was positive in approximately 35% of the tests performed (Figure 1). Females had a higher percentage of positive results compared to males in all age groups; after the age of 40, this difference was even greater.

### 3.2. Autoantibody positivity in terms of specialities and hospitals

#### 3.2.1. Rheumatoid factor

Autoantibody positivity rates differed according to the specialties that requested the test. Information related to other specialties is given in supplement 1. The top five branches that requested the RF test were family medicine (38.2%), internal medicine (15.6%), physical therapy and rehabilitation (12.5%), orthopedic surgery (7.1%), and rheumatology (5.4%). RF positivity at those departments was as follows: family medicine 10.4%, internal medicine 18.3%, physical therapy and rehabilitation 18.2%, orthopedic surgery 16.0%, and rheumatology 26.8%. RF test positivity at different levels of care was as follows: primary care 10.3%, secondary and tertiary referral centers 17.0%, university hospitals 26.9%, and private hospitals 18.5%.

#### 3.2.2. Anti-CCP

The top three branches that requested the anti-CCP test were rheumatology (43.7%), physical therapy and rehabilitation (22.9%), and internal medicine (17.9%). Anti-CCP positivity at these departments was as follows: rheumatology 42.1%, physical therapy and rehabilitation 38.3%, and internal medicine 45.7%. Anti-CCP test positivity at different levels of care was as follows: primary care 27.1%, secondary and tertiary referral centers 41.6%, university hospitals 37.3%, and private hospitals 56.7%.

### 3.3. RF and anti-CCP positivity in RA patients

There were 435,735 people for whom either M05 or M06 ICD codes were entered twice at least 3 months apart. While 189,116 (43.4%) patients had RF positivity, 122,029/205,683 (59.3%) patients had anti-CCP positivity.

### 3.4 RA diagnosis in autoantibody-positive people

**Figure 2:**
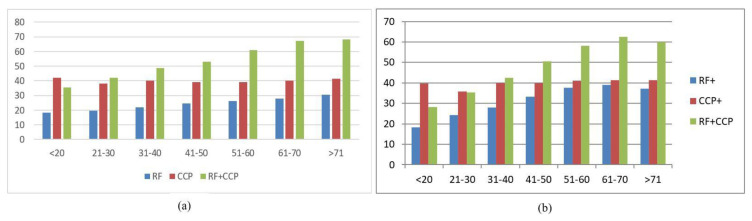
(a) (females). RA diagnosis percentage within the RF, anti-CCP, and both autoantibodies positive people with respect to age. (b) (males). RA diagnosis percentage within the RF, anti-CCP, and both autoantibodies positive people with respect to age.

RF positivity was detected in 1,333,160 people. This number was 262,254 for anti-CCP positive patients and 179,832 for concomitant RF and anti-CCP positive patients. RA was diagnosed in 27.8% of RF-positive people and in 39.73% of anti-CCP-positive people. This rate increased to 56.6% for both RF and anti-CCP positive people. The RA diagnosis rate increased in all age groups for both RF positivity and RF plus anti-CCP positivity. The risk was 18.3% for the RF positive and 32.9% for the RF plus anti-CCP positive group for the youngest group; on the other hand, it increased to 32.3% for the RF positive and 65.9% for the RF plus anti-CCP positive group in the oldest group. The diagnosis of RA was seen in approximately 40% of all age groups of anti-CCP patients and did not change with age or sex. Females tended to have more frequent RA diagnoses in RF positive and RF plus anti-CCP positive groups (Figures 2a and 2b).

## 4. Discussion

Significant data on RF and anti-CCP were obtained from the online database covering the entire country. The demand for tests per year, the rates of repeated tests in the same year, the positivity rates of the tests, and their distribution by age and sex were determined separately according to various departments and different levels of care. The prevalence of RA based on ICD-10 codes was also reported for the first time in the present study (0.70%).

In the study, data from across the country were reviewed and it was determined that RF tests were requested from 5.7% and anti-CCP tests from 1.9% of the population by their physician annually. It was found that frequent RF and anti-CCP requests in this way entail a significant cost, particularly since 15% of RF tests are requested again within the same year. In a previous study, the cost of RF testing for the diagnosis of RA was investigated. It was revealed that it cost £405 per positive result. £708.75 was spent per case of seropositive RA [19], but there was no information about anti-CCP testing. As a result of being a nationwide study, our costs were lower compared to that study’s results. The latter was a single-center study; thus selection bias might have occurred.

When RF was requested by the doctor in the community, positivity was found in 14.7% of the population. While this reaches the lowest rate when requested by family physicians (approximately 10%), it reaches the highest rate when requested by rheumatologists in university hospitals. Regarding RF, we note that a significant portion of the tests are probably requested by primary care physicians for screening purposes. Similar rates are obtained for orthopedic surgeons as well. These results show that the RF test is used as a screening test by doctors who are not experts in this area. Previous community-based studies showed the percentage of the presence of RF positivity in the general population to be between 2.8% and 21.6% [3,13]. Our study is the largest study in the literature that includes all age groups and the prevalence of RF positivity was 13.49%. Consistent with the literature, women had more RF positivity [14,17]. Autoimmunity is generally encountered more in women; hormone profile could be the main reason for this disparity between males and females. On the other hand, the positivity rates in the anti-CCP test are much higher (35.0%), and anti-CCP tests are rarely requested by primary care doctors. The prevalence of anti-CCP in the general population has been studied in limited studies [3–5]. In those studies, the anti-CCP positive rate in the healthy population ranged from 0.8% to 5.5%. All of these studies primarily involved healthy people who had no joint symptoms. The most likely explanation is that anti-CCP is mainly studied in people who had joint symptoms, presenting to rheumatology, physiotherapy and rehabilitation, and internal medicine physicians, who ask for anti-CCP tests more frequently, and the positivity rates of those patients are as high as 42%–45%. It is also observed that the positivity rate in the tests ordered by these doctors is higher for RF. All these findings suggest that RF, which is more accessible, is mainly used by family physicians and orthopedic physicians for screening purposes. It is found to be used in a more specific and effective manner by musculoskeletal specialists.

In the present study, it is seen that RF test positivity increases with age in the population; positivity, however, in anti-CCP tests has a similar rate regardless of age. Like in our study, there was a tendency for positive RF results as the tested population got older [15,20]. However, there is no information related to anti-CCP positivity and age relation in the literature. The effect of the simultaneous positivity of RF and CCP on diagnosis is controversial [21]. In our study, 25% of all RF and anti-CCP tests were positive; likewise, in RF, it is more common in women and increases with age. Considering the relatively similar percentage of anti-CCP positivity among the age groups, the difference is likely due to the distribution of RF positivity. Consistent with our study, previous studies have indicated that anti-CCP positivity has a higher potency by detecting patients with RA compared to RF positivity [21]. The subgroup analysis of this metaanalysis claimed that RF and concomitant anti-CCP positivity had little contribution to the diagnosis compared to either anti-CCP or RF positivity. However, in our study, concomitant antibody positivity presents the greatest risk of diagnostic RA concerning either RF or anti-CCP positivity alone. The inconsistency could be because the total number of patients that had both anti-CCP and RF measurements was quite low compared to our study (4590 versus 797,089). As explained earlier, our study population was more likely to have a diagnosis of RA, which could contribute to the differences in our findings.

In the Turkish RA prevalence studies, it was found that prevalence ranged from 0.32% to 1.01% [22–26]. In the present study, all data in Türkiye were reviewed. When evaluated according to ICD-10 codes, the frequency of RA was found to be 0.7%. When assessing the ICD-10 codes, the condition to enter similar codes 2 or more times was observed. However, care needs to be taken when commenting on ICD-10 codes. It should always be remembered that the diagnosis for these patients may have been entered incorrectly and differently in some cases. In the literature, M05 and M06 ICD codes were compared with autoantibody test results and positivity rates between 57.3% and 74.1% were found [27,28]. Nevertheless, 59% of patients diagnosed with RA had positive anti-CCP antibodies, similar to the general literature.

Our study has some strengths and limitations. It has the largest population in which RF and anti-CCP have been investigated in the literature to date. It also gives important information about very young and old groups that have not studied much before. Its retrospective design may have caused some missing data. People with ICD 10 codes that were entered for the RA at least twice with an interval of 3 months were accepted to have RA. However, it is possible that some patients did not have follow-up or applied once during the study period. Although anti-CCP tests are generally required from reference centers, the lack of a laboratory system with national RF-specific standards is one of the limitations of the study. Finally, the history of smoking, which is known to be associated with RF and anti-CCP positivity, and the lack of data on certain other diseases, such as other autoimmune disorders or chronic hepatitis, may have increased the prevalence of autoantibody positivity, especially for the RF tested population.

Finally, countrywide data were examined in the present study. RF and anti-CCP positivity rates were observed in the community when requested by the doctor. The effects of age, sex, department, and hospitals, which can influence this positivity, have been revealed. Based on these findings, it will be possible to create algorithms to request RF and anti-CCP tests in a smarter way.

## Supplement

**Supplement Table 1:** The number of rheumatoid factor (RF) positive patients and the corresponding positivity rates across various medical specialties

Speciality	RF (+) person	RF (+) ratio (%)
Family Medicine	436272	10,4
Internal Medicine	314095	18,3
Physiotherapy and Rehabilitation	250165	18,2
Orthopedics	124796	16,0
Rheumatology	159806	26,8
Emergency Medicine	53924	13,9
Pediatric	41963	14,5
Neurology	41284	19,6
Public Health	10832	5,7
General Surgery	26032	16,9

Abbrevation: RF: rheumatoid factor

## Figures and Tables

**Table:** Comparison of rheumatoid factor and anti-CCP antibodies.

Features	Rheumatoid factor	Anti-CCP

Mean age (SD)	47.7 (7.9)	47.9 (7.5)

Sex (female)	67.4%	75.4%

Tests per year (n)	3,479,518	1,183,607
Test per year/total adult population (%)	5.7	1.9

Cost (TL)		
Annual	23,625,653	8,674,868
Per RA diagnosis	149.5	55
Per sero + RA diagnosis	500	183

Repeated test in same year n (%)	403,402 (15.8)	14,518 (5.2)

Positivity when requested in general population (%)	14.7	35.0
i. Age 20–30	11.2	32.9
ii. Age 41–50	14.3	35.3
iii. Age >71	20.8	35.6

Regarding specialities’ requests n (%)and positivity (%)	Family medicine (38.2), 10.4	Rheumatology (43.7), 42.1
	PTR (12.5), 18.2	
	Internal medicine (15.6), 18.3	PTR (22.9), 38.3
	Orthopedic surgery (7.1), 10.6 Rheumatology (5.4), 26.8	Internal medicine (17.9), 45.7

Positivity regarding hospitals (%)		
i. Primary care	10.3	27.1
ii. 2nd and 3rd referral centers	17.0	41.6
iii. University hospitals	26.9	37.3
iv. Private hospitals	18.5	56.7

Positivity in RA patients regarding ICD-10 code (M05 and M06) (%)	43.4	59.3
